# Adherence of traffic-related particles to human red blood cells *in vivo*

**DOI:** 10.1183/23120541.00767-2025

**Published:** 2026-03-16

**Authors:** Jonathan Grigg, David Wertheim, Simon Crust, Emanuel Jeldes, Rodger Duffin, Barbara A. Maher, Norrice M. Liu

**Affiliations:** 1 Centre for Genomics and Child Health, Blizard Institute, London, UK; 2 Faculty of Engineering, Computing and the Environment, Kingston University, Surrey, UK; 3 Centre for Inflammation Research, University of Edinburgh, Edinburgh, UK; 4 Lancaster Environment Centre, University of Lancaster, Lancaster, UK

## Abstract

**Background:**

There is indirect evidence that inhaled traffic-related particulate matter (PM) penetrates into the human circulation. Since nanoparticles readily adhere to red blood cells (RBCs) *in vitro*, we sought to determine whether a mechanism of systemic transport of translocated traffic-related particles is *via* adherence to RBCs *in vivo*.

**Methods:**

Adult volunteers were exposed to traffic-related emissions from a main road for 1 h. Volunteers were also exposed to emissions wearing a FFP2 mask. Exposure to black carbon PM was assessed by portable aethalometer. The mean area (μm^2^) of adherent black PM per RBC was determined from unstained blood smears from 3000 cells by light microscopy. Particle composition was determined by scanning transmission electron microscopy and energy-dispersive X-ray analyses. The capacity of diesel exhaust particles to adhere to human RBCs *in vitro* was determined, and RBCs were examined after intratracheal instillation of diesel exhaust particles to a mouse model.

**Results:**

Exposure to traffic-related emissions increased personal black carbon PM (n=12, p=0.001 *versus* baseline). Exposure increased the area of particles adherent to RBCs (p<0.001 *versus* baseline), and this was reduced by wearing a FFP2 mask (p=0.002 *versus* no mask). Traffic exposure increased the abundance of metal-bearing nanoparticles associated with RBCs. Diesel exhaust particles adhered to RBCs *in vitro* in a dose-dependent manner. Particles were found adherent to circulating RBCs after intratracheal instillation of diesel exhaust particles.

**Conclusion:**

Adhesion of traffic-related PM to RBCs is a systemic transport mechanism. Quantification of particles on RBCs is a putative practical biomarker of inhaled dose.

## Shareable abstract

**Translocated traffic-related carbonaceous and metal-bearing particles are transported *in vivo via* adhesion to RBCs. Wearing a FFP2 mask reduces systemic PM dose.** https://bit.ly/4u5n0Qu

## Introduction

A putative mechanism for the adverse health effects of traffic-related particulate matter (PM) (a complex mix of particles from exhaust emissions, and brake and tyre wear) is that the smallest fractions of inhaled particles translocate into the bloodstream. Translocation of inhaled PM has been reported in healthy volunteers after inhalation of gold nanoparticles (*i.e.* particles with at least one dimension <100 nm) [1], and white spots of light compatible with carbonaceous particles are reported in human blood samples exposed to femtosecond pulsed laser illumination [2]. Furthermore, exogenous, metal-bearing nanoparticles have been identified not only in the frontal cortex and brainstem of the brain [3], and myocardial mitochondria in the heart [4], but also in ventricular red blood cells (RBCs) [5] and serum [5].

One potential systemic transport mechanism for translocated inhaled PM previously studied for delivering therapeutic nanoparticles, is *via* adherence to circulating RBCs, a phenomenon called “RBC hitchhiking” [6]. In studies using animal models, adherence of polystyrene nanoparticles to RBCs *in vitro* protects against their rapid elimination, *i.e.* 5% of RBCs with adherent nanoparticles in circulation after 12 h *versus* >99.9% unbound particles eliminated within 2 min [7]. Since adherent particles on circulating RBCs have previously been reported in rats after intratracheal instillation of ultrafine (<100 nm) carbon black [8], we sought evidence for systemic transport of translocated particles *via* adhesion to RBCs after exposure of adult volunteers to road traffic-related emissions.

## Methods

### Exposure to traffic emissions

Healthy, nonsmoking adults working in London (UK) were recruited between May and August 2024. All volunteers provided informed written consent (Queen Mary University of London ethics approval reference number QME23.0041). On days without rain, volunteers stayed in an office building in Whitechapel (London) with a heating, ventilation and air conditioning system. After 4 h, participants walked to a nearby main road (Whitechapel Road or Commercial Road, London) and remained within 10 m of traffic for 1 h (supplementary figure S1). After roadside exposure to traffic-related emissions, volunteers returned to the air-conditioned office and remained for a further 1 h. In a subgroup of volunteers, exposure to traffic-related emissions was repeated on a different day wearing an FFP2 mask (Kolmi OP-air PRO oxygen) walking to, and standing next to, the main road. Personal exposure to black carbon-bearing PM was assessed by a portable aethalometer (microAeth AE51; Aethlab, CA, USA) with flow rate set at 100 mL·min^−1^. Mean black carbon concentration (µg·m^−3^·min^−1^) was determined for each volunteer for baseline, traffic-related emissions exposure, and post-exposure periods.

### RBCs after exposure to road traffic emissions

Either venous or capillary blood (2–5 mL) was obtained from volunteers after 4 h in the office environment (baseline), immediately after 1 h exposure to road traffic (exposure), and 1 h after return to the office environment (post-exposure). Blood samples were collected into sterile plastic tubes spray-coated with lithium heparin (Becton Dickinson, NJ, USA) in the air-conditioned office. Blood films were generated using a standard protocol [9]. Slides with blood films were air dried in a climate-controlled laboratory with no fixing or staining before imaging. As previously reported for assessment of carbonaceous (*i.e.* black) PM in airway and placental macrophages [10, 11], the study's *a priori* outcome was area of adherent carbonaceous PM per RBC (PM-RBC area). Briefly, RBCs were imaged using light microscopy (×60) with a standardised image acquisition pattern. The area of carbonaceous PM expressed as mean μm^2^ per RBC was determined for each sample by image analysis from unselected 3000 RBCs using ImageJ software 1.50i (National Institutes of Health, USA), as described previously [10, 11]. Although our previous study of combustion particles in placental phagocytes analysed 1000 cells per sample [11], we chose to examine a total of 3000 RBCs *a priori* since experimental animal studies have suggested that <1% of ultrafine particles deposited in the alveoli pass into the bloodstream [12]. Other variables measured were the total number (n) of carbonaceous (black) particles from 3000 RBCs, and the total number of RBCs with one or more adherent carbonaceous particles from 3000 RBCs. Analysis was blinded to exposure.

### Imaging and elemental analysis of particles

Blood smears were fixed with Sigma Aldrich Hemacolor fixing solution, which is methanol-based (Merck Life Science, UK). Slides were dipped in the solution and air dried while supported vertically. The unstained slides without coverslips were imaged using an Olympus OLS4100 reflection confocal microscope (Olympus, Japan) in fine mode using a ×50 or ×100 objective lens. For scanning electron microscopy (SEM) imaging, glass slides prepared as described earlier were sputter-coated with a gold palladium alloy of 10–15 nm thickness. Imaging was performed with a Zeiss EVO 50 SEM using Smart SEM service pack 5 software. SEM images were obtained using secondary electron imaging with an acceleration voltage of 10 kV and working distance of 8.5 mm. Images were captured where the cells appeared to be RBCs as identified by their discoid appearance. The elemental composition of particles identified in SEM imaging was analysed using an energy-dispersive X-ray spectroscopy (EDX) system (Oxford Instruments Nanotechnology Tools AZtecLive Advanced 6.1 service pack 2 software with Ultim Max 65 detector). In a different scanning and transmission electron microscopy (STEM) facility, RBCs from a positive control (*i.e.* RBCs with diesel exhaust particles (DEP) added *in vitro*), and from a pre-exposure and post-exposure sample from a volunteer were mounted on electron microscopy support grids (gold), which were then carbon-coated to provide a stable, conductive surface facilitating high-resolution imaging of these delicate biological samples, and minimising charging effects. We used transmission electron microscopy (FEI Titan3 Themis 300, X-FEG S/TEM with S-TWIN objective lens, monochromator (energy spread ∼0.25 eV) and multiple high-angle annular dark field/annular dark field/bright-field STEM detectors, operated at 300 kV), and energy-dispersive X-ray analysis (EDX, FEI Super-X 4-detector system) to examine the location, shape, size, and, perhaps most critically in terms of toxicity and biological impact, elemental composition of nanoscale electrodense nanoparticles associated with the RBCs. To limit beam-induced damage, a probe current of 60 pA was used to acquire the elemental data. To ensure that the nanoparticles identified and analysed were representative, grids and grid areas were randomly selected and methodically scanned.

### Adhesion of diesel exhaust particles to RBCs *in vitro*

DEP from industrial forklifts (standard reference material 2975; National Institute of Standards and Technology, USA), at 0.5 μg·mL^−1^, 1 μg·mL^−1^ and 5 μg·mL^−1^ were added to 100 µL heparinised human blood for 1 h at 37°C. Blood films were generated and imaged under light microscopy as described earlier for determination of mean PM-RBC area per cell from 3000 cells.

### Intratracheal instillation of diesel exhaust particles

Female C57Bl6 mice aged 10 weeks were anaesthetised using 4% isoflurane and either 50 μL 0.9% sterile saline (vehicle) or DEP (Standard Reference Material 2975, NIST) in suspension (50 μL at 1 mg·mL^−1^) administered to the lungs by intratracheal instillation, as described previously [1]. Whole-blood samples (10 μL), taken *via* the tail vein, were collected at baseline immediately prior to instillation, and 5 min, 30 min, 60 min and 24 h post-instillation. Blood smears onto sterile microscope slides at each time point were done immediately following sample collection for analysis of mean PM-RBC area from 3000 cells, as described earlier. Analysis was blinded to exposure group. All experiments were performed according to the Animals (Scientific Procedures) Act 1986 (UK Home Office project licence number PP5472015).

### Statistical analysis

Data are summarised as median (interquartile range (IQR)) since data were not normally distributed. Comparisons between multiple datasets were done using Friedman (paired data) or Kruskal–Wallis (unpaired data) one-way ANOVA with Dunn's multiple comparisons test. Comparison between two datasets was done using the Mann–Whitney test. Analyses were done using Prism 10.2.1 for Windows (GraphPad Software, CA, USA). Correlations were done by Spearman's rank correlation (R_s_). Results are considered significant at p<0.05. Since there were no previous data on PM-RBC area, no power calculation was done for the human exposure study. Practical considerations limited numbers studied to a maximum of 12 volunteers. Since the animal study sought to confirm the presence of adherent particles, to reduce animal use, number was limited to three animals.

## Results

### Exposure to traffic emissions

**FIGURE 1:**
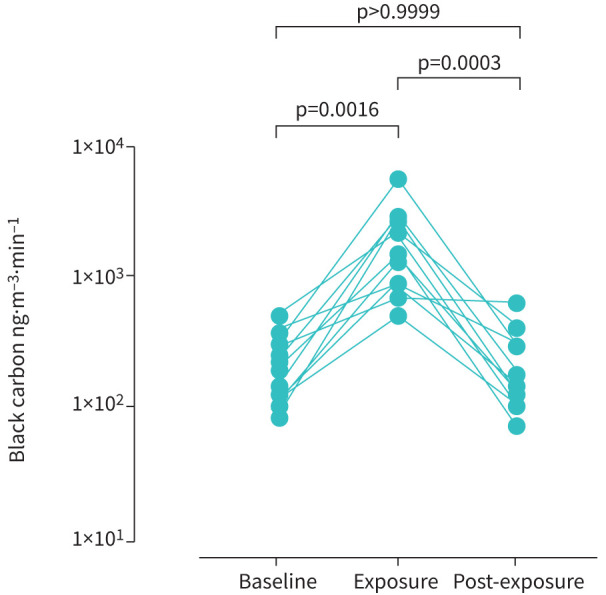
Exposure of healthy volunteers (n=12) to black carbon measured by portable aethalometer. Data are expressed as mean ng·m^−3^·min^−1^. Comparisons to baseline by Friedman one-way ANOVA with Dunn's multiple comparisons test.

12 adult volunteers were recruited: median (IQR) age 39 (32–44) years; five males and seven females. Standing next to the main road for 1 h increased personal exposure to black carbon compared with baseline (n=12, median (IQR) 1806 (863–2887) ng·m^−3^·min^−1^ *versus* 229 (114–369) ng·m^−3^·min^−1^, p=0.0016; figure 1).

**FIGURE 2:**
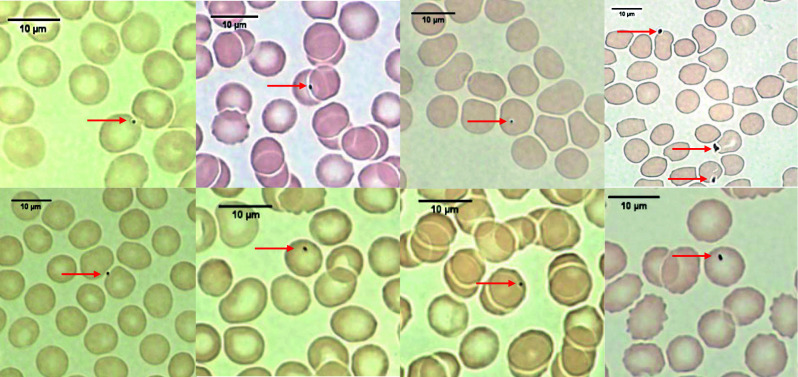
Representative light microscopy images of carbonaceous (black) particles adherent to red blood cells (red arrows) from healthy volunteers. Each image is from a different adult volunteer. Scale bars=10 μm.

**FIGURE 3:**
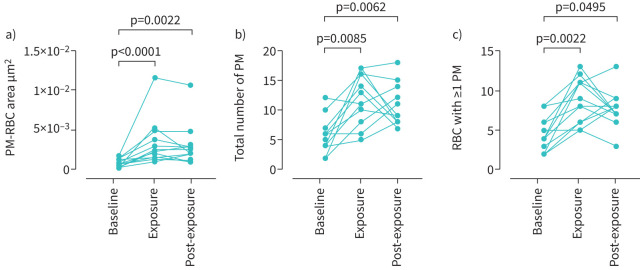
Effect of *in vivo* exposure to traffic-related emissions on the adhesion of carbonaceous particulate matter (PM) to red blood cells (RBCs) (n=12). a) Area of carbonaceous PM adherent to red blood cells (PM-RBC; mean area of carbonaceous PM inclusions per RBC); b) total number of adherent carbonaceous PM; c) total number of RBCs with at least one adherent carbonaceous PM. Data are from analysis of 3000 RBCs per adult volunteer. Comparisons with baseline by Friedman one-way ANOVA with Dunn's multiple comparisons test.

In all volunteers, irregular carbonaceous (black under light microscopy) particles adherent to RBCs (PM-RBC) were found after exposure to traffic-related emissions (figure 2). Free carbonaceous PMs were very rarely observed between RBCs. Exposure to road traffic significantly increased PM-RBC area compared with baseline (median (IQR) 8.0×10^−4^ (3.0×10^−4^–1.4×10^−3^) μm^2^ per RBC *versus* 2.3×10^−3^ (1.5×10^−3^–4.6×10^−3^) μm^2^ per RBC, n=12, p<0.0001; figure 3a), which remained increased at 1 h post-exposure (p=0.0022 *versus* baseline; figure 3a).

**FIGURE 4:**
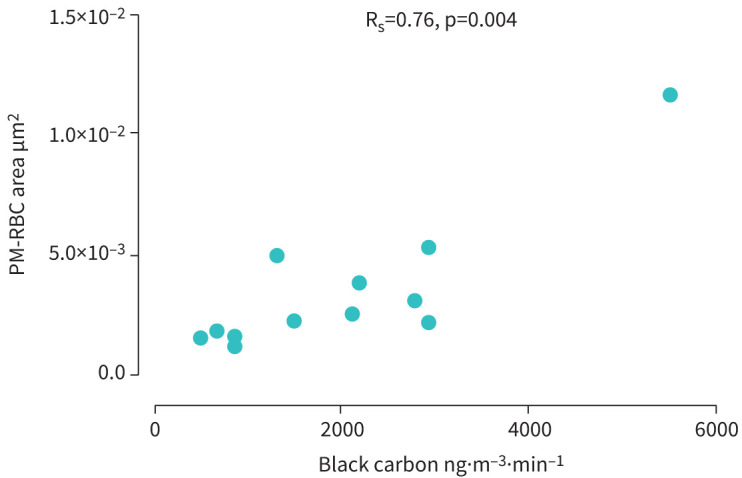
Correlation of mean personal black carbon during traffic measured by aethalometer, and mean area of adherent carbonaceous particles per red blood cell (PM-RBC area). R_s_: Spearman's rank correlation.

Exposure to traffic emissions also increased other measures of particle adhesion. First, the total number (n) of adherent particles per volunteer in 3000 RBCs increased after exposure to road traffic (baseline *versus* exposure; median (IQR) 5.5 (4.0–6.7) *versus* 12 (8.5–16), p=0.008; figure 3b), and at 1 h post exposure (*versus* baseline p=0.006; figure 3b). Second, the number of RBCs from 3000 cells with one or more adherent particles per volunteer increased after road traffic exposure (baseline *versus* exposure; median (IQR), 4.0 (2.2–6.0) *versus* 8.5 (6.0–11), p=0.002; figure 3c), and at 1 h post-exposure (*versus* baseline p=0.049; figure 3c). Overall, there was a positive correlation (R_s_=0.76, p=0.004) between personal black carbon concentrations during exposure to road traffic, and PM-RBC area assessed immediately after 1 h exposure (figure 4).

### Wearing a FFP2 mask

**FIGURE 5:**
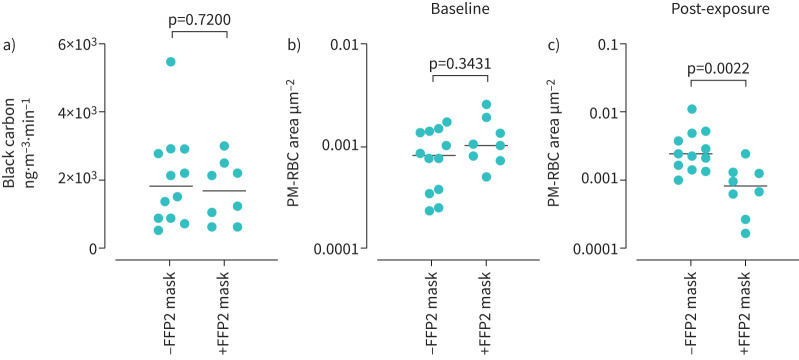
Effect of wearing a FFP2 mask during exposure to traffic-related emissions on the area of carbonaceous particles adherent to red blood cells (RBC-PM area, μm^2^ per RBC). Data are from eight subjects who wore a mask (+FFP2) and are compared with 12 subjects exposed to traffic-related emissions without wearing a mask and studied on a different day (−FFP2). a) Personal black carbon exposure (median; ng·m^−3^·min^−1^) during 60 min of traffic-related emission exposure, measured by portable aethalometer; b) baseline (pre-exposure) PM-RBC area; c) post-exposure PM-RBC area. Comparisons by Mann–Whitney test. Bars represent the median.

Exposure to traffic-related emissions was done in eight volunteers wearing an FFP2 mask (+FFP2). Results were compared with data from 12 volunteers exposed without a mask (−FFP2, described earlier). There was no significant difference in personal exposure to traffic-related black carbon PM (median (IQR) −FFP2 1806 (863–2887) ng·m^−3^·min^−1^ *versus* +FFP2 1674 (728–2420) ng·m^−3^·min^−1^, p=0.7200; figure 5a). There was no difference in baseline PM-RBC (−FFP2 *versus* +FFP2, 8.0×10^−4^ (3.0×10^−4^–1.4×10^−3^) μm^2^ per RBC *versus* 1.0×10^−3^ (7.3×10^−4^–1.7×10^−3^) μm^2^ per RBC, p=0.3431; figure 5b). Wearing a FFP2 mask during exposure to road traffic-related emissions significantly reduced PM-RBC area (−FFP2 *versus* +FFP2 2.3×10^−3^ (1.5×10^−3^–4.6×10^−3^) *versus* 8.4×10^−4^ (3.5×10^−4^–1.3×10^−3^) μm^2^ per RBC, p=0.0022; figure 5c).

### Adherence of diesel exhaust particles to RBCs *in vitro*

DEP readily adhered to human RBCs *in vitro* in a dose-dependent manner. Compared with control (*i.e.* no added DEP), PM-RBC area increased significantly with added DEP concentrations ≥1.0 µg·mL^−1^ (median (IQR), 0 *versus* 1.0 µg·mL^−1^, n=10, 1.0×10^−3^ (4.6×10^−4^–1.5×10^−3^) μm^2^ per RBC *versus* 4.5×10^−3^ (3.3×10^−3^–9.7×10^−3^) μm^2^ per RBC, p=0.0055; supplementary figure S2A). A similar dose-dependent increase was found for the total number of adherent PM per 3000 RBCs (supplementary figure 2B), and the number of RBCs with one or more adherent carbonaceous PM per 3000 RBCs (supplementary figure 2C).

### Composition of particles

**FIGURE 6:**
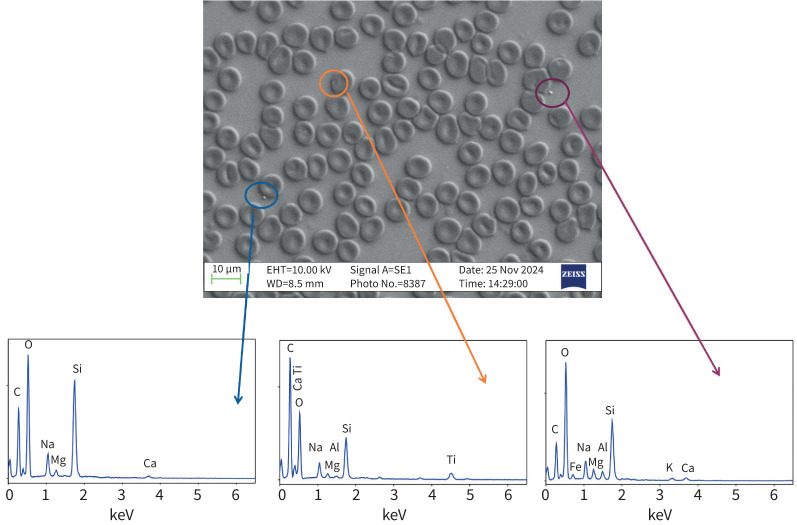
An example scanning electron microscopy image with three areas of particles associated with red blood cells circled. The corresponding spectrum for each particle is shown below the image.

**FIGURE 7:**
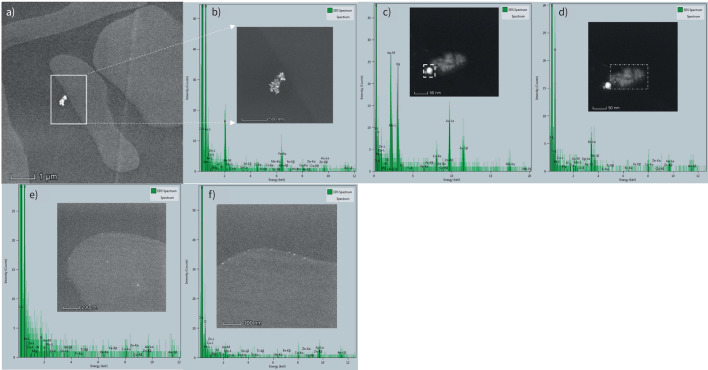
Scanning and transmission electron microscopy and energy-dispersive X-ray analyses of red blood cell (RBC)-associated nanoparticles (NPs). a) Cluster of electron-dense NPs close to RBC margin; b) NP cluster (white box in a) particle morphology and elemental composition (containing Fe>Cu, Ti>Cr, Zn, Sn (Mo, from electron microscope sample holder); c) rounded NP (in dashed white box) containing Ag>Cu>Al>Fe>Si; d) NP aggregate with indistinct “fluffy” edges, containing Sn>Cu>Si>Fe; e) dispersed NPs on the surface of RBCs, containing Si>Cu, Al>Mg>Ti, Cr, Fe, Ni, Zn, Sn; f) NPs around the edges of RBCs, containing Cu>Fe>Sn.

Scanning electron microscopy of RBCs after exposure to traffic-related emissions *in vivo* showed particles closely adjacent or adherent to RBCs (figure 6), with EDX spectra from 11 particles showing peaks from a range of metals and metalloids, including silicon, calcium, iron, sodium, magnesium, aluminium and titanium. STEM and EDX analyses were additionally performed in a separate facility, in order to characterise the nano-sized particles associating with the surface of RBCs (figure 7a–f). ∼60 particles/particle clusters were subjected to EDX, encompassing >200 individual particles. Metal-bearing nanoparticles were readily observable, occurring both as clusters comprising ∼10–100 nanoparticles (figure 7a, b, d) and as single or several nanoparticles, distributed across the surface of the cells (figure 7c, e) and, notably, along the cell edges (figure 7f). Compared to the positive control sample (*i.e.* RBC with DEP added *in vitro*) and the “pre-exposure” baseline sample, metal-bearing nanoparticles were seen in greatest abundance in the post-exposure sample. Some nanoparticles display rounded, even spherical shapes (figure 7c); others appear slightly elongate or have “fluffy”/indistinct outlines (figure 7d). Individual nanoparticles range in size from ∼3 to 125 nm: those around the RBC edges ∼5–10 nm (figure 7e, f). Aggregates of nanoparticles range from ∼150 to 220 nm. In terms of the metal composition of the observed nanoparticles, common to all three samples was the presence of silicon, iron, copper and silver. Of these, iron and silicon were the most abundant. Additional elements found in the nanoparticles were titanium (positive control and post-exposure samples), calcium, fluorine and sulfur (pre-exposure sample), chromium and nickel (post-exposure sample), and phosphorus and tin (positive control sample).

### Intratracheal instillation of diesel exhaust particles

**FIGURE 8:**
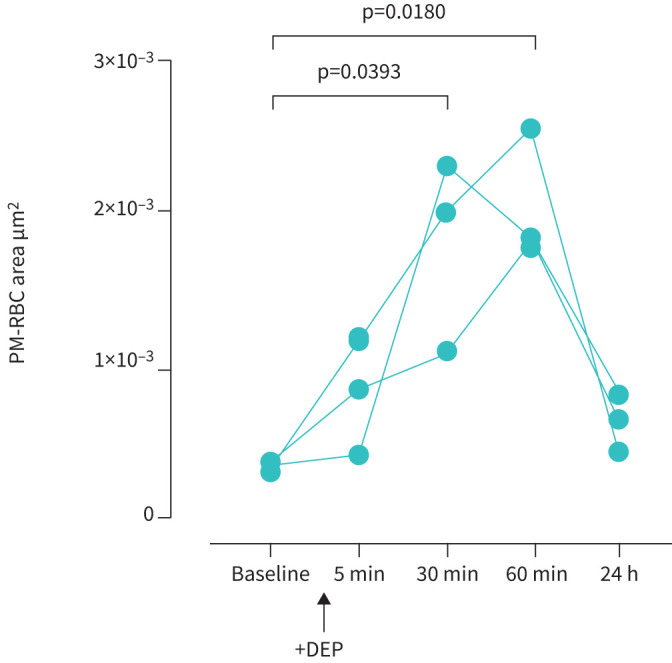
Change in carbonaceous particulate matter (PM) adherent to red blood cells (PM-RBC area/RBC, μm^2^ per RBC) after intratracheal instillation of diesel exhaust particles (DEP) to mice (n=3). Comparisons with baseline by Friedman ANOVA with Dunn's multiple comparisons test.

In mice (n=3), carbonaceous (black) particles were observed adherent to RBCs after intratracheal instillation of DEP. The morphology of PM adherent to RBC was similar to that in volunteers exposed to traffic-related emissions (supplementary figure S3). Compared with baseline, PM-RBC area increased at 30 min (median (IQR) 3.4×10^−4^ (2.8×10^−4^–3.6×10^−4^) μm^2^ per RBC *versus* 1.9×10^−3^ (1.0×10^−3^–2.2×10^−3^) μm^2^ per RBC, p=0.039; figure 8), and at 60 min after intratracheal instillation (p=0.018; figure 8). The PM-RBC area returned to baseline by 24 h (figure 8).

## Discussion

In this study we sought to test the hypothesis that adhesion to RBCs is a mechanism of transport of translocated traffic-related particles in the systemic circulation. In adult volunteers, we found particles consistent with carbonaceous and metal-bearing, traffic-related particles adherent to RBCs *in vivo*. Furthermore, the area of adherent particles increased after short-term exposure to road traffic emissions. These findings are compatible with Bongaerts *et al.* [2], who detected white light spots, suggestive of carbonaceous PM, in human blood samples using femtosecond pulsed laser light-stimulation. Although the proportion of RBCs with adherent PM (median 0.26%) after exposure to traffic-related emissions is low, assuming RBC concentrations between 4.2 and 6.2 million cells·mL^−1^, between 10 900 and 16 120 RBC·mL^−1^ are transporting PM.

It is unlikely that the observed and analysed particles are artefactual. First, the morphology of adherent particles *in vivo* is similar to fossil-fuel and other traffic-derived PM found in human airway macrophages, and human placental macrophages [10, 11, 13, 14]. Second, adherent carbonaceous particles in unstained blood smears cannot be endogenous structures such as platelets, which are colourless refractile structures in unstained smears, or Howell–Jolly bodies: nuclear remnants in RBCs and visible after staining as purple spots. Third, since blood smears were done in an air-conditioned laboratory, contamination by ambient particles is unlikely. Fourth, as reported previously [15], we found that DEP readily adhered to RBCs in a dose-dependent manner *in vitro*, and we detected black particles adherent to RBCs in the blood of mice after intratracheal instillation of DEP. Finally, electron microscopy and elemental analyses showed nanoparticles, both as clusters and dispersed particles, directly associated with RBC surfaces and cell edges. The range of metals/metalloids associated with these nanoparticles includes nonphysiological species (*e.g.* aluminium, titanium, tin, nickel) in association with others (*e.g.* iron, copper, silicon, chromium and zinc) which are characteristic of traffic-derived particulate matter <10 μm in aerodynamic diameter, both from exhaust and nonexhaust sources [16, 17]. A known anthropogenic metal, silver [18], where present, occurred in association with copper and molybdenum, suggesting brake and/or tyre wear as its possible source [19].

Consistent with the present study's light microscopy findings, RBC-associated nanoparticles were most abundant in a post-exposure blood sample under advanced imaging. Since there was a correlation between personal traffic-related black carbon exposure and quantity of particles on RBCs, we speculate that analysis of blood smears by light microscopy is a promising way of assessing the effect of exposure mitigation. Indeed, wearing a FFP2 mask during exposure to road emissions significantly reduced the quantity of particles adherent to circulating RBCs. Although the beneficial effect of a FFP2 mask has previously been modelled *in vitro* using aerosolised sodium chloride [20], the present study provides the first *in vivo* evidence that a FFP2 mask reduces inhaled dose of traffic-related PM.

There are limitations to this study. Since there was no unexposed control group, we did not assess the contribution of longer-term exposure to ambient pollutants on baseline (pre-exposure) RBC-PM. Therefore, further studies are needed to fully define variables associated with RBC-PM area, including the effects of long-term exposure, age, sex and lung diseases such as cystic fibrosis associated with impaired airway particle removal [21]. We speculate that differences in PM-RBC kinetics explain the differences between individuals in the present study. For example, while most volunteers demonstrated a decline in PM-RBC an hour after acute pollution exposure, some showed a continuous increase in PM-RBC. Although the possibility of an unknown confounder cannot be completely excluded, these would not explain either the increase in PM-RBC after traffic emission exposure, or the reduction in PM-RBC area wearing a FFP2 mask, or the increase in PM-RBC area after intratracheal DEP in the mouse model. Furthermore, the appearance of particles on RBCs immediately at 60 min after *in vivo* exposure to traffic-related emissions is compatible with a study using ultrafine technetium-99m-labelled carbon particles, which detected radioactivity in the blood of volunteers within minutes after inhalation [22], and with the study of Shimada *et al.* [8], who observed in a mouse model black particles adhering to RBCs within 8 min of intratracheal instillation of ultrafine carbon black. A further study limitation is that we did not determine the effect of particle adhesion to RBCs on RBC function *per se*. However, Pan *et al.* [23] reported that adherence of nanoparticles to RBCs *in vitro* sensitises RBCs to both mechanical and oxidative stress, and Zhang *et al.* [24] reported that adherence of fossil-fuel PM to mouse RBCs *in vitro* deforms RBCs. Finally, the fate of particles adherent to RBCs *in vivo* is unclear. Brenner *et al.* [6] hypothesised that adhesion of particles to RBCs increases particulate transfer to endothelial cells lining capillaries, and further *in vitro* studies are therefore needed to assess whether the small fraction of RBCs with adherent PM induce biologically relevant effects in target cells (*e.g.* endothelial cells). Whether adhesion to RBCs is the main systemic transport mechanism for translocated traffic-related PM, and whether RBCs transport other particles such as nanoplastics, remains unclear.

In conclusion, this study found that translocated traffic-related carbonaceous and metal-bearing particles are transported *in vivo via* adhesion to RBCs, and that wearing a FFP2 mask reduces systemic PM dose.

## Data Availability

Prism files and individual images of cells supporting the key findings of this study are available by emailing j.grigg@qmul.ac.uk.

## Supplementary material

**Please note:** supplementary material is not edited by the Editorial Office, and is uploaded as it has been supplied by the author. Supplementary material 00767-2025.SUPPLEMENT

## References

[1] Miller MR, Raftis JB, Langrish JP, et al. Inhaled nanoparticles accumulate at sites of vascular disease. ACS Nano; 2017; 11: 4542–4552. doi:10.1021/acsnano.6b08551 28443337 PMC5444047

[2] Bongaerts E, Lecante LL, Bové H, et al. Maternal exposure to ambient black carbon particles and their presence in maternal and fetal circulation and organs: an analysis of two independent population-based observational studies. Lancet Planet Health 2022; 6: e804–e811. doi:10.1016/S2542-5196(22)00200-5 36208643 PMC9553674

[3] Maher BA, Ahmed IAM, Karloukovski V, et al. Magnetite pollution nanoparticles in the human brain. Proc Natl Acad Sci USA 2016; 113: 10797–10801. doi:10.1073/pnas.1605941113 27601646 PMC5047173

[4] Maher BA, González-Maciel A, Reynoso-Robles R, et al. Iron-rich air pollution nanoparticles: an unrecognised environmental risk factor for myocardial mitochondrial dysfunction and cardiac oxidative stress. Environ Res 2020; 188: 109816. doi: 10.1016/j.envres.2020.109816 32593898 PMC7306213

[5] Lu D, Luo Q, Chen R, et al. Chemical multi-fingerprinting of exogenous ultrafine particles in human serum and pleural effusion. Nat Commun 2020; 11: 2567. doi: 10.1038/s41467-020-16427-x 32444803 PMC7244483

[6] Brenner JS, Mitragotri S, Muzykantov VR. Red blood cell hitchhiking: a novel approach for vascular delivery of nanocarriers. Annu Rev Biomed Eng 2021; 23: 225–248. doi: 10.1146/annurev-bioeng-121219-024239 33788581 PMC8277719

[7] Chambers E, Mitragotri S. Prolonged circulation of large polymeric nanoparticles by non-covalent adsorption on erythrocytes. J Control Release 2004; 100: 111–119. doi: 10.1016/j.jconrel.2004.08.005 15491815

[8] Shimada A, Kawamura N, Okajima M, et al. Translocation pathway of the intratracheally instilled ultrafine particles from the lung into the blood circulation in the mouse. Toxicol Pathol 2006; 34: 949–957. doi: 10.1080/01926230601080502 17178695

[9] US Centers for Disease Control and Prevention Division of Parasitic Diseases and Malaria. Blood specimens – specimen processing. Date last accessed: 21 February 2025. www.cdc.gov/dpdx/diagnosticprocedures/blood/specimenproc.html

[10] Kulkarni N, Pierse N, Rushton L, et al. Carbon in airway macrophages and lung function in children. N Engl J Med 2006; 355: 21–30. doi: 10.1056/NEJMoa052972 16822993

[11] Liu NM, Miyashita L, Maher BA, et al. Evidence for the presence of air pollution nanoparticles in placental tissue cells. Sci Total Environ 2021; 751: 142235. doi: 10.1016/j.scitotenv.2020.142235 33181987

[12] United States Environmental Protection Agency. Particle pollution exposure. Date last accessed: 28 February 25. www.epa.gov/pmcourse/particle-pollution-exposure

[13] Liu NM, Chen Y, Miyashita L, et al. The presence of air pollution particulate matter in cryopreserved placental tissue cells. ERJ Open Res 2021; 7: 00349-2021. doi: 10.1183/23120541.00349-2021 34435037 PMC8381264

[14] Nwokoro C, Ewin C, Harrison C, et al. Cycling to work in London and inhaled dose of black carbon. Eur Respir J 2012; 40: 1091–1097. doi: 10.1183/09031936.00195711 22362851

[15] Nemmar A, Zia S, Subramaniyan D, et al. Interaction of diesel exhaust particles with human, rat and mouse erythrocytes *in vitro*. Cell Physiol Biochem 2012; 29: 163–170. doi: 10.1159/000337597 22415085

[16] Beddows DCS, Harrison RM, Gonet T, et al. Measurement of road traffic brake and tyre dust emissions using both particle composition and size distribution data. Environ Pollut; 2023; 331: 121830. doi: 10.1016/j.envpol.2023.121830 37211228

[17] Maher BA, Ahmed IAM, Davison B, et al. Impact of roadside tree lines on indoor concentrations of traffic-derived particulate matter. Environ Sci Technol 2013; 47: 13737–13744. doi: 10.1021/es404363m 24215538

[18] Wang YF, Huang KL, Li CT, et al. Emissions of fuel metals content from a diesel vehicle engine. Atmos Environ 2003; 37: 4637–4643. doi 10.1016/j.atmosenv.2003.07.007

[19] Garg BD, Cadle SH, Mulawa PA, et al. Brake wear particulate matter emissions. Environ Sci Technol 2000; 34: 4463–4469. doi 10.1021/es001108h

[20] Maciejewska M, Przybyła M, Szczurek A. Aerosol penetration study for FFP2 half masks regarding protection against diesel particles in underground mines. J Occup Environ Hyg 2023; 20: 480–492. doi: 10.1080/15459624.2023.2238022 37656966

[21] Liu NM, Miyashita L, Sanak M, et al. Prostaglandin E_2_ and phagocytosis of inhaled particulate matter by airway macrophages in cystic fibrosis. J Cyst Fibros 2021; 20: 673–677. Doi: 10.1016/j.jcf.2020.11.010 33250436

[22] Nemmar A, Hoet PHM, Vanquickenborne B, et al. Passage of inhaled particles into the blood circulation in humans. Circulation 2002; 105: 411–414. doi: 10.1161/hc0402.104118 11815420

[23] Pan DC, Myerson JW, Brenner JS, et al. Nanoparticle properties modulate their attachment and effect on carrier red blood cells. Sci Rep 2018; 8: 1615. doi: 10.1038/s41598-018-19897-8 29371620 PMC5785499

[24] Zhang Y, Zhong L, Zhan J, et al. Unraveling potential causative components for the deleterious effect of atmospheric fine particulate matter on red blood cells. Environ Sci Technol 2024; 58: 21954–21965. doi: 10.1021/acs.est.4c06657 39601440

