# Supplementary material for: Adherence of traffic-related particles to human red blood cells in vivo
Source: ERJ Open Res. 2026 Mar 16;12(2):00767-2025. doi: 10.1183/23120541.00767-2025 (PMC12991022; doi:10.1183/23120541.00767-2025)
Supplement: Supplementary file 1 [file 00767-2025.SUPPLEMENT.pdf]

Online Supplement: Transport of traffic-related carbonaceous particulate matter in humans by red blood cells

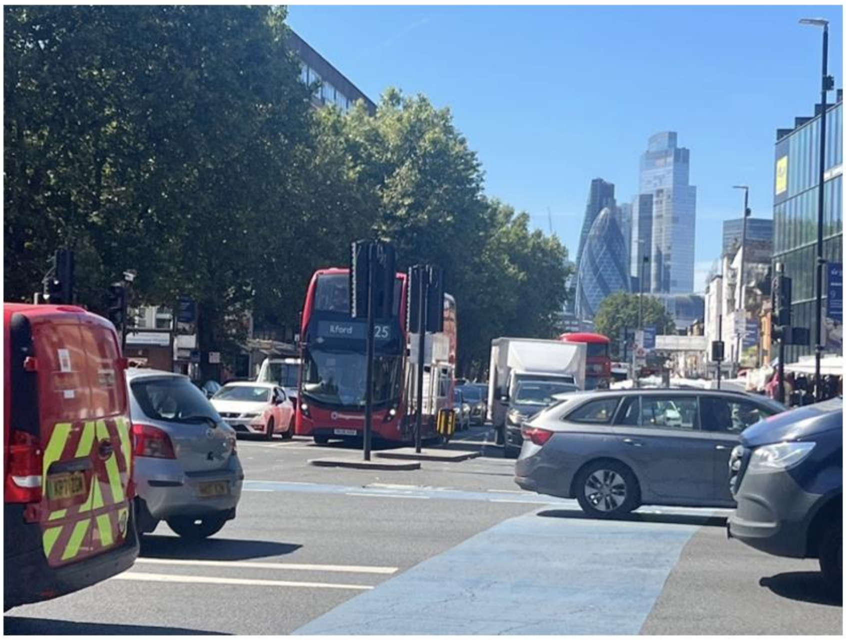

Supplement, Figure 1; Traffic on Whitechapel Road (London, UK)

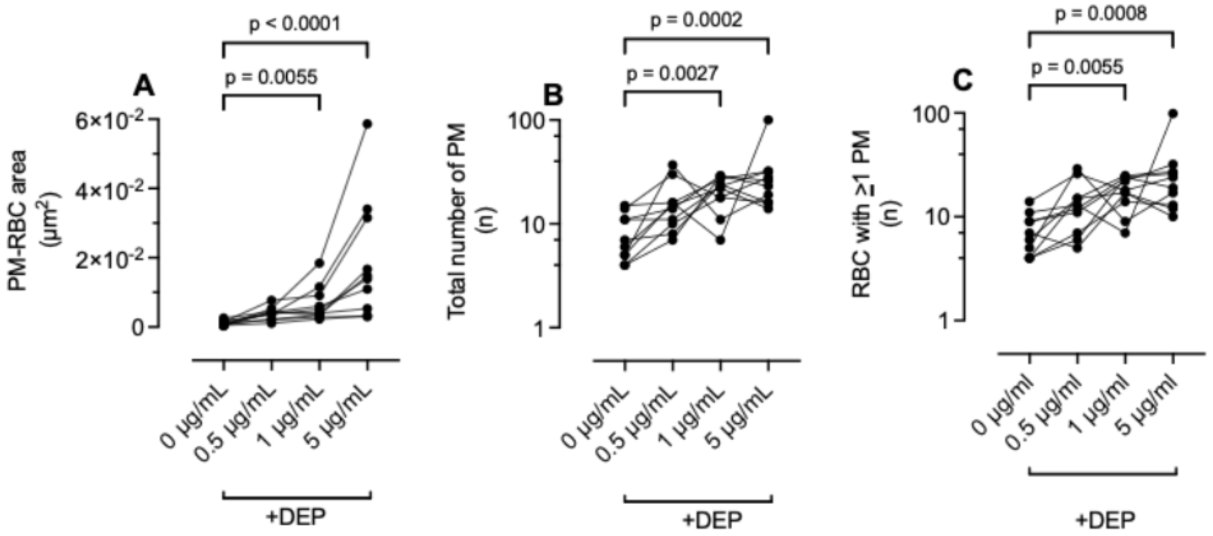

Supplement, Figure 2; Adhesion of diesel exhaust particles (DEP) to human red blood cells (RBCs) *in vitro*, n=10; **A**) area of adherent carbonaceous particulate matter (PM) particles (PM-RBC area per RBC); **B**) total number of adherent carbonaceous particles from 3000 RBCs; **C**) total number of RBCs with one or more adherent carbonaceous particles from 3000 RBCs. Comparisons by Friedman one-way ANOVA with Dunn's multiple comparisons test.

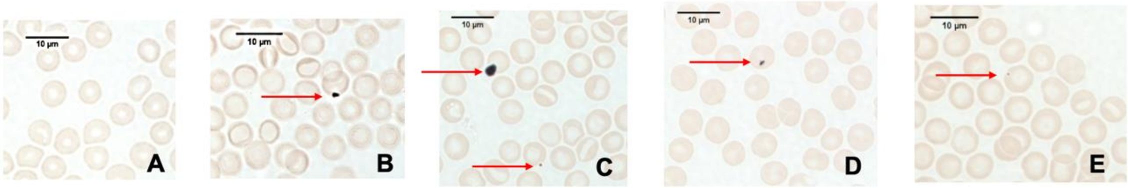

Supplement, Figure 3; Representative images of black particulate matter inclusions on red blood cells from a single mouse, A) before intratracheal instillation of diesel exhaust particles, B) 5 min post instillation, C) 30 min post instillation, D) 60 min post instillation, E) 24 h post instillation.
